# The impact of type 2 diabetes on tendon material properties and locomotor function in the *db*/*db* mouse

**DOI:** 10.1113/EP093650

**Published:** 2026-02-15

**Authors:** James P. Charles, Estella Chen, Jeff Hart, Andrea Bell, Brendan Geraghty, Roger W. P. Kissane

**Affiliations:** ^1^ Department of Musculoskeletal and Ageing Science University of Liverpool Liverpool UK; ^2^ Cica Biomedical Ltd. Knaresborough UK; ^3^ School of Biomedical Sciences University of Leeds Leeds UK

**Keywords:** diabetes mellitus, kinematics, musculoskeletal model, tendon material properties

## Abstract

Obesity affects billions of individuals world‐wide and brings with it greater susceptibility to secondary conditions, like that of type 2 diabetes mellitus (T2DM). One of the most effective therapeutic treatment strategies for obesity and T2DM is exercise, yet both populations present with severe exercise intolerance. Understanding the mechanisms driving this impaired locomotor/exercise function may help to better design therapeutic treatment strategies and exercise training regimes. Here, using a diabetic mouse model (*db*/*db*), we explore the functional decline of tendons and the subsequent impact on locomotor function. Firstly, we present measurements of *ex vivo* viscoelastic and tensile properties for the Achilles tendon in diabetic and wild‐type mice. The results show that while the viscoelastic properties (tendon hysteresis and stress‐relaxation) do not change, the diabetic tendons present with impaired uncrimping of the collagen fibres, resulting in an increased strain at physiological stresses. Next, we built mouse‐specific hindlimb musculoskeletal models incorporating body mass and tendon material properties to predict the impact of changing physiology on locomotor function. Diabetic mouse models showed significant increases in muscle activation and metabolic output, alongside shifts to a less spring‐like and more negative work‐dominated brake‐like function in the more tendinous muscles of the hindlimb. Together, our experimental and modelling data indicate that *db*/*db* mice generate larger degrees of muscle power to move the same distance, driven by the combined effects of increased body mass and tendon compliance. Alleviating either of these physiological issues may help recover patients’ exercise tolerance and improve their overall quality of life.

## Abstract (highlights)

- One of the most effective treatment strategies for type 2 diabetes mellitus is exercise, yet these patients present with severe exercise intolerance: what are the mechanisms driving this impaired exercise tolerance?
- Using a mouse model of type 2 diabetes, viscoelastic properties of tendons do not change, but the uncrimping of collagen fibres is impaired, resulting in increased tendon strain at physiological stresses. Using mouse‐specific musculoskeletal modelling we highlight the consequence of elevated body mass and altered tendon mechanics on muscle recruitment, linking it to the elevated metabolic cost of locomotion.

## 1 INTRODUCTION

Obesity is a global health problem affecting billions of individuals worldwide. This continually expanding number places a mounting strain on health and social care systems, with obesity‐related conditions costing the United Kingdom's National Health Service £11.4 billion each year. Chronic obesity predisposes individuals to secondary conditions like Type 2 diabetes mellitus (T2DM), which may further elevate the likelihood of injury to both soft (e.g. tendons and ligaments) (Xu et al., 2024) and hard tissues (e.g. bone) (Janghorbani et al., 2007; Shanbhogue et al., 2016; Vestergaard, 2007), further reducing the quality of life. One of the most effective therapeutic treatment strategies for obesity and T2DM is exercise, yet both populations present with severe exercise intolerance (Anderson et al., 2025; Poitras et al., 2018). Understanding the mechanisms driving this impaired locomotor/exercise function may help to better design therapeutic treatment strategies and exercise training regimes.

T2DM presents with an interesting dichotomy of mechanical and chemical signalling. Firstly, the elevated body mass increases the loading on the musculoskeletal system (Charles et al., 2025), which may lead to adaptive increases in muscle mass, altered tendon properties (such as increased elastic modulus and decreased hysteresis) (Wiesinger et al., 2015) and increased bone mineral density (Lekkala et al., 2019). However, the prolonged hyperglycaemia, accumulation of advanced glycation end products (AGEs) and chronically elevated inflammation associated with T2DM (Suriano et al., 2021) have all been shown to decrease muscle quality (Garnham et al., 2020), reduce elastic modulus of tendons (Connizzo et al., 2014; Fox et al., 2011) and make bones weaker (Lekkala et al., 2019). The complex balance of these adaptive–maladaptive signals and the consequences of these changes on locomotor function are still poorly understood.

Tendons function as biological springs during locomotion to store and release elastic energy and improve locomotor performance and efficiency (Biewener, 1998; Blazevich & Fletcher, 2023; Maganaris & Paul, 1999; Uchida et al., 2016). Across a range of studies using diabetic rodent models, the described pathological changes in Achilles tendon material properties are conflicting (Supporting information, Appendix Figure A1). For instance, some studies show evidence of a reduced elastic modulus (or stiffness) (Boivin et al., 2014; de Oliveira et al., 2011), some present no change (Volper et al., 2015), while others show an increase (Silva et al., 2017) (Appendix Figure A1A). Similar differences in response are seen for maximum tension (Appendix Figure A1B) and specific strain (Appendix Figure A1C). This ambiguity appears to present not only in animal models but also across T2DM patient populations (Zellers et al., 2021a). The Achilles tendon of T2DM patients has been shown to increase (Petrovic et al., 2018), decrease (Evranos et al., 2015; Guney et al., 2015), or present with no discernible differences in stiffness (Couppe et al., 2016). These contradictory findings may be due to methodological approaches used (Zellers et al., 2021a), making the mechanisms driving any altered mechanical properties equally unclear. While the trend in tendon mechanical properties is not clear from the literature, there is a wealth of evidence for these patients presenting with significantly elevated cost of transport (Petrovic et al., 2018), which may be one of the critical underpinnings of the populations severe exercise intolerance. Understanding the underlying physiology that results in increased metabolic demand might aid the development of targeted therapeutic treatment strategies that improve the exercise tolerance of this population.

Subsequently, we set out to characterise the material properties of tendons in response to diabetes and understand the consequences of these changes on locomotor performance. First, we completed an overview of viscoelastic and tensile mechanical tests on the isolated Achilles tendon of a diabetic mouse. We hypothesised that the chronic AGE accumulation (Xu et al., 2024) and systemic inflammation (Suriano et al., 2021) would result in an increase in hysteresis, indicative of a less efficient elastic material (hypothesis 1a). Furthermore, we also hypothesised that the diabetic tendons would have a reduced elastic modulus and UTS, ultimately making the tendons more susceptible to damage (hypothesis 1b) (Lake et al., 2023).

Next, we sought to understand the functional consequence of elevated body mass and altered tendon mechanics on functional performance. To do this, we used a previously validated musculoskeletal model and simulation of trotting locomotion in the mouse hindlimb (Charles et al., 2018, 2016, 2024) to predict changes in musculotendon function in response to increased body mass and altered tendon stiffness (i.e., a virtual diabetic mouse model). Based on previous work studying the impacts of tendon compliance on individual muscle dynamics using musculoskeletal models and simulations (Bates et al., 2025; Sellers et al., 2010; Uchida et al., 2016), we hypothesised that a reduction in tendon stiffness in the diabetic mice would result in higher activations and metabolic power outputs in more tendinous muscles during trotting, and shifts away from acting like energy‐efficient springs (hypothesis 2).

## 2 METHODS

### 2.1 Ethical approval

All experimental procedures were performed in accordance with the UK Animals (Scientific Procedures) Act 1986 and approved by the University of Liverpool's Animal Welfare and Ethical Review Committee (AWC0215, PPL: P81E9540D). This work conforms to the ethical requirements outlined by the journal, and is presented in accordance with guidelines for animal work (Grundy, 2015; Percie du Sert *et al.*, 2020).

### 2.2 Animals

Six male control (C57B6) mice (24.95 ± 0.64 g, 11–12 weeks old, Charles River, Kent, UK) and 14 male diabetic (BKS.Cg‐*Dock7^m^ *+/+ *Lepr^db^*/J) mice (40.45 ± 4.08 g, 11–12 weeks old, The Jackson Laboratory, ME, USA) were used in this study. Animals were housed under a 12‐h light–dark cycle at 21°C and had ad libitum access to food and water. Mice were culled by Schedule 1 approved methods with a rising concentration of CO_2_. Power calculations performed using mean differences and SD values from tensile data of *db*/*db* mice, taken from Boivin et al. (2014) (α, 0.05; power, 0.80; two‐tailed), suggest that a minimum of *n* = 5 per group would provide sufficient power to detect significant differences, with our sample size exceeding this.

#### 2.2.1 Tendon material properties

First, the diameter of the control (wt, *n* = 6) and diabetic (*db*/*db n* = 8) Achilles tendons were measured under a light microscope, with 10 evenly spaced measurements taken between the insertion of the tendon (calcaneus) and the aponeurosis approaching the belly of the triceps surae (across approximately 5 mm of tendon). The tendons were then attached to the Instron Universal Testing System (68SC‐5, Instron, Wycombe, UK) fitted with a 50 N load cell (2530‐50N, Instron). Samples were submerged in a phosphate buffer solution at 37°C and allowed to thermoequilibrate for 15 min. A pre‐load of 0.01 N was applied, and five load‐unload preconditioning cycles up to 0.5 N at 100% min^−1^ rate (Fung, 2013; Readioff et al., 2022). Viscoelastic tests were performed to examine the effect of strain rate on energy dissipation (hysteresis), stress‐relaxation and a ramp to failure. The tendons underwent four loading cycles at 10% min^−1^, 100% min^−1^ and 1000% min^−1^, with 6 min rest intervals between each strain rate. The stress–relaxation behaviour was determined after a ramp to 0.5 N at 100% min^−1^ and held for 1000 s. The final test was a ramp to failure completed at 100% min^−1^. Load‐displacement data were converted to stress (Equation 1) and strain (Equation 2) required to calculate elastic modulus (Equation 3). Energy loss during cyclical loading was presented as percentage lost (Equation (4), (5), (6)).

(1) $$\sigma =\frac{F}{\mathrm{CSA}}$$

σ is stress in Pa, *F* is the measured force in newtons, and CSA is the cross‐sectional area of the tendon, calculated from the average radius (*r*) of the tendon (π*r* ^2^).

(2) $$\varepsilon =\frac{\Delta L}{L_{0}}$$

where ε is strain, Δ*L* is the change in length in mm and *L*_0_ is the starting length.

(3) $$\mathrm{Elastic}\;\mathrm{Modulus}=\frac{\Delta \sigma }{\Delta \varepsilon }$$

Elastic modulus was taken during the most linear portion of the ramp to failure, as well as between 0.2 and 0.3 MPa, which represents the physiological range of stress that muscles can generate (Kissane & Askew, 2024; Matson et al., 2012).

(4) $$U=\sum_{k=1}^{N}\frac{1}{2}\times \left(\sigma_{k-1}+\sigma_{k}\right)\times \Delta \varepsilon_{k}$$

where *U* is the stored energy in Pa, *N* is the resolution of the trapezoid partition, and Δε*_k_* is the length of the *k*th interval (Δε*_k_* = ε*_k_* − ε*_k_*_−1_).

(5) $$\mathrm{Hysteresis}\;=U_{\mathrm{loading}}-U_{\mathrm{unloading}}$$

where *U*_loading_ and *U*_unloading_ represent the stored energy during the loading and unloading of the tendon, respectively.

(6) $$\mathrm{Energy}\;\mathrm{Lost}\;\;\left(\%\right)=\frac{\mathrm{Hysteresis}}{U_{\mathrm{loading}}}\times 100$$

#### 2.2.2 Building a diabetic musculoskeletal model

A previously published musculoskeletal model of the mouse hindlimb and pelvis (Charles et al., 2018, 2016) with muscle‐specific force‐velocity properties (Charles et al., 2024) was used as the basis for the model used here. This mouse model was scaled to match the body masses of the individual mice used to derive tendon material properties (see above), with the percentage tendon strain at maximum muscle isometric force also changed to match the experimentally derived data from these specific mice (assuming an isometric stress of 0.3 MPa). All other aspects of the musculoskeletal system (i.e., body segment lengths and muscle force‐generating properties) were assumed to be consistent between the control and diabetic groups. This resulted in three modelled groups: Control (*n* = 6, subject‐specific mass and tendon properties from the experimental control mice), Obese (*n* = 8, subject‐specific body mass from the experimental diabetic mice with the average control tendon properties) and Diabetic (*n* = 8, subject‐specific body mass and tendon material properties from the experimental diabetic mice). The Obese group was included here to isolate the impacts of tendon properties from body mass on predictions of musculotendon function.

In each of these model variants, motion data and external forces (i.e., ground reaction forces) from Charles et al. (2018) were used to generate simulations of a single gait cycle of trotting locomotion using OpenSim MOCO (Dembia et al., 2020) and the MocoInverse function. This predicted the muscle forces and activations necessary to satisfy the external forces during this motion in each model variant. Metabolic probes (Umberger, 2010) were applied to each muscle in the model to predict individual and total muscle metabolic power output. Dimensionless functional indices (Charles et al., 2024, 2022; Kissane et al., 2023; Lai et al., 2019), which classify muscles as acting as struts, springs, motors or brakes (depending on the magnitude and timing of force and work production throughout the gait cycle) were calculated for individual muscles to quantify the impact of tendon stiffness on functional behaviour (see previous work for details of their calculation). These outputs are reported for the medial gastrocnemius (MG), lateral gastrocnemius (LG), tibialis anterior (TA), iliacus (ILI) and vastus lateralis (VL) muscles. These muscles were chosen as they are either largely tendinous muscles (MG, LG and TA), and therefore likely to be sensitive to changes in tendon properties, or heavily active during the gait cycle at more proximal joints (ILI and VL).

##### Statistical analyses

All data processing and figures were plotted using Igor Pro 8 (V8.0.4.2). All statistics (Shapiro–Wilk, Student's *t*‐test, linear regression and one‐way ANOVA/Kruskal–Wallis) were completed using SPSS Statistics 28 (28.0.1.1, IBM Corp., Armonk, NY, USA), where the threshold for statistical significance was set to *P *< 0.05. *Post hoc* comparisons were made using the Bonferroni correction with a threshold for statistical significance set at *P *< 0.05. Statistical differences in stress–relaxation curve data were calculated at each data point using one‐dimensional statistical parametric mapping (1D SPM) (Pataky, 2012) in MATLAB (R2018a, MathWorks, Natick, MA, USA). All data are presented as means ± standard deviation.

## 3 RESULTS

### 3.1 Diabetic mice have significantly greater body mass but similar muscle masses compared to wild‐type mice

**FIGURE 1:**
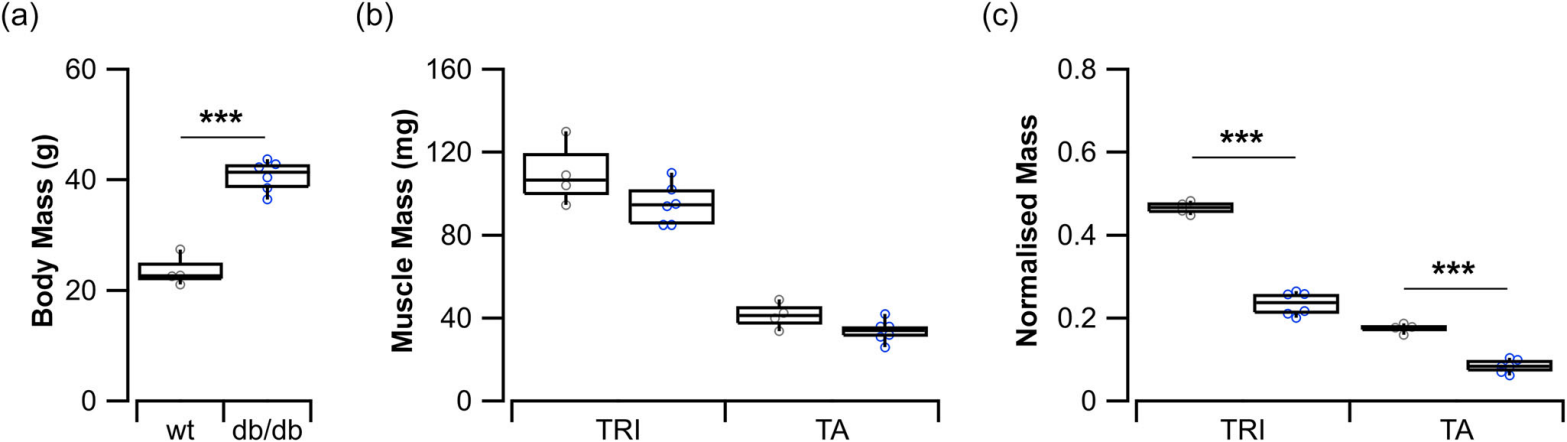
Body morphometrics. (a) Body mass was significantly elevated in the diabetic (*db*/*db*) mice compared to controls (wt). (b, c) The absolute muscle mass does not significantly differ across the two groups (b), while the relative muscle mass (normalised to body mass) does significantly differ (c). wt (*n* = 4), *db*/*db* (*n* = 6). ***P < 0.001. TA, tibialis anterior; TRI, triceps surae muscle group.

Despite the significantly greater body mass in the diabetic mice (40.7 ± 2.8 vs. 23.5 ± 2.7 g, *t*_8_ = −9.599, *P *< 0.001, Figure 1a), the muscles that insert via the Achilles tendon (Lee & Elliott, 2019), the soleus, medial and lateral gastrocnemius (triceps surae group; TRI) were not significantly different compared to controls (95.2 ± 9.8 vs. 109.4 ± 15.0 mg, *t*_8_ = 1.833, *P* = 0.104, Figure 1b). Similarly, the tibialis anterior (TA) was not significantly different (33.8 ± 5.5 vs. 41.3 ± 6.3 mg, *t*_8_ = 1.993, *P* = 0.081, Figure 1b). However, when normalised to body mass, both the TRI muscle group (0.24 ± 0.03 vs. 0.47 ± 0.02, *t*_7.873_ = 16.544, *P *< 0.001, Figure 1c) and TA (0.08 ± 0.02 vs. 0.18 ± 0.01, *t*_8_ = 9.778, *P *< 0.001, Figure 1c) of the diabetic mice were significantly smaller compared to controls. Achilles tendon cross‐sectional area was significantly larger in the diabetic compared to the control mice (wt 1.07 ± 0.14 mm^2^ vs*. db*/*db* 1.94 ± 0.83 mm^2^, *t*_12_ = −2.52, *P* = 0.027).

### 3.2 Diabetes does not alter viscoelastic properties but leads to altered tensile‐tendon material properties

**FIGURE 2:**
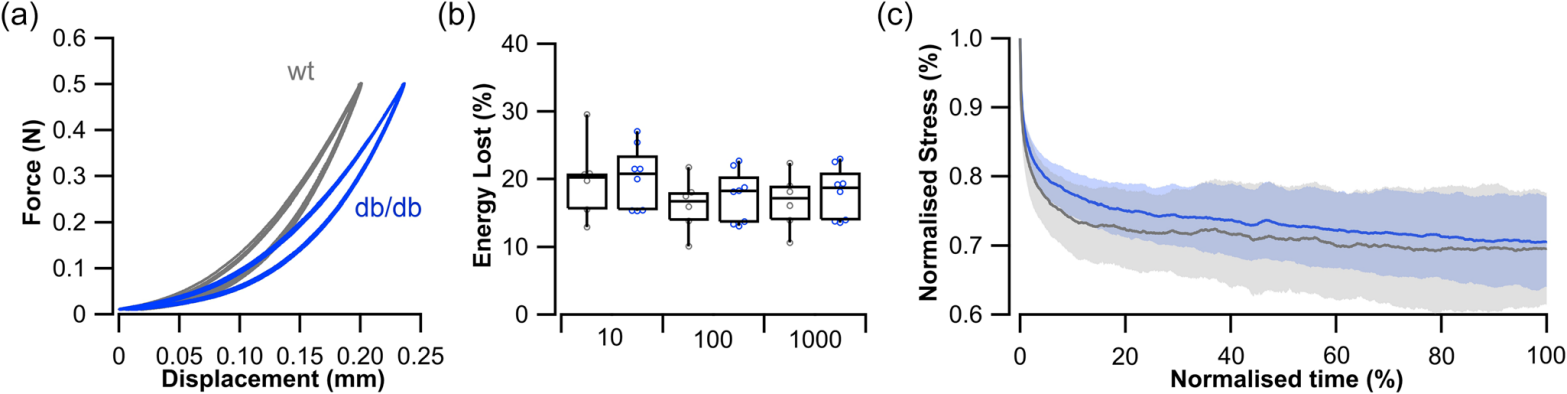
Viscoelastic properties of the diabetic tendon. (a) The Achilles tendon underwent cyclical loading at three different strain rates 10, 100 and 1000% min^−1^. (b) There was no significant difference in the hysteresis (dissipated energy) between the control (wt; *n* = 6) and diabetic (*db*/*db*; *n* = 8) tendons across the three strain rates. (c) Stress–relaxation experiments show that across a 1000 s hold, there was no significant difference in the decline in stress, as determined by one‐dimensional statistical parametric mapping (1D‐SPM). Continuous line represents the mean ± SD (shaded area).

The viscoelastic properties of the Achilles tendon were assessed using cyclical loading at different strain rates (Figure 2a,b) and a ramp–hold stress–relaxation run (Figure 2c). There was no significant difference in the proportion of energy dissipated at 10% min^−1^ (*t*_12_ = −0.125, *P* = 0.902, Figure 2a,b), 100% min^−1^ (*t*_12_ = −0.640, *P* = 0.534, Figure 2b) and 1000% min^−1^ (*t*_12_ = −0.590, *P* = 0.566, Figure 2b). Similarly, the stress–relaxation curves showed there to be no statistically significant difference in the rate of force relaxation behaviour between the control and diabetic tendon (Figure 2c) as determined by 1D SPM (Supporting information, Appendix Figure A2).

**FIGURE 3:**
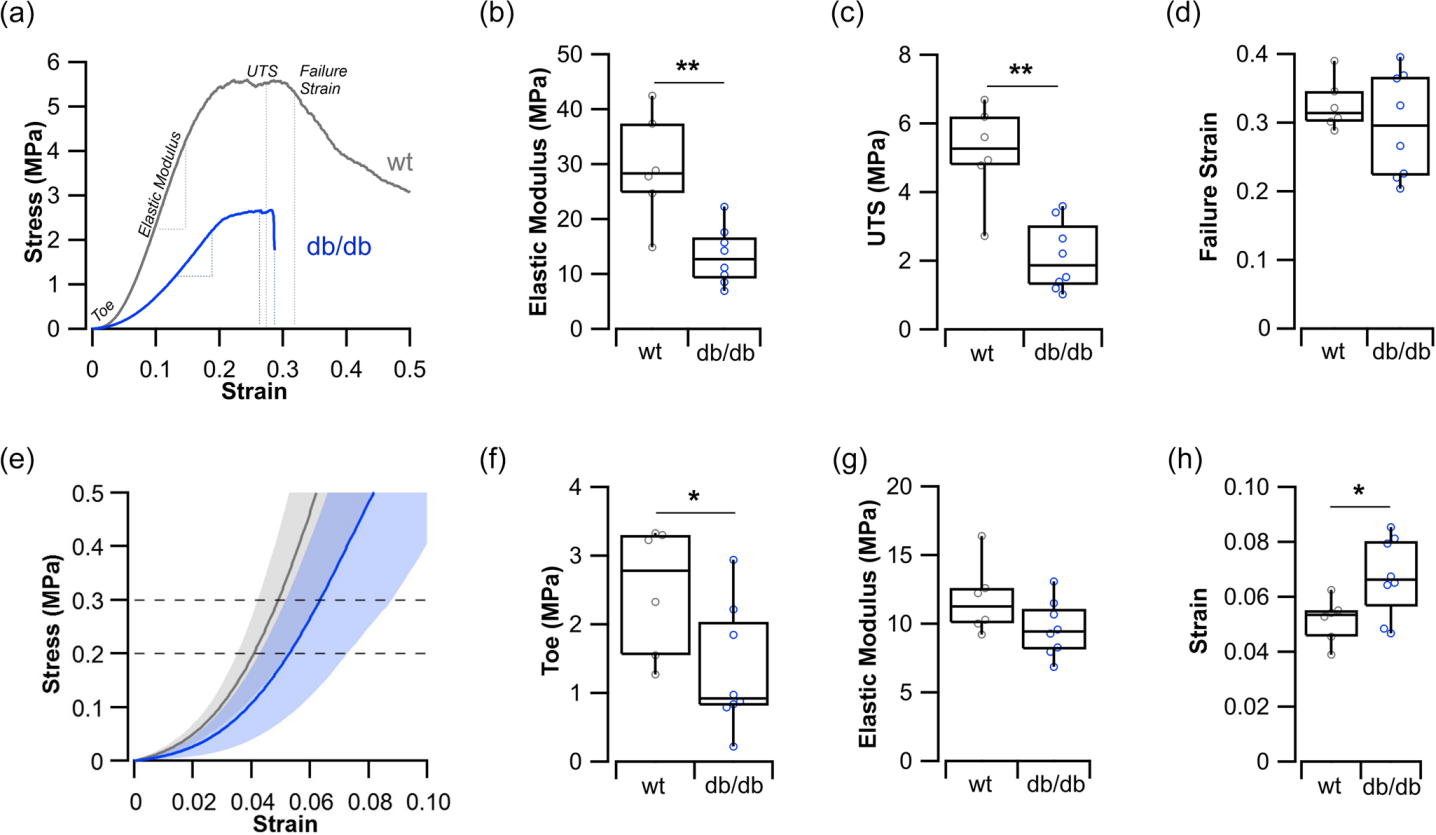
Achilles tendon material properties. (a) Representative stress–strain curve for an Achilles tendon from the wild type (wt) and diabetic (*db*/*db*) mouse. (b) Elastic modulus measured from the linear phase of the stress–strain curve shows the diabetic mice to have a significantly lower stiffness. (c) These mice also present with a significantly lower ultimate tensile strength (UTS). (d) However, the strain at which the tendons fail does not significantly differ. (e) The physiological range of stresses that a tendon might be exposed to during locomotion depends on the levels of activation of the muscles. (f) The toe region of the curve (i.e., 2% strain) presents with a significantly lower ratio of stress‐to‐strain in the diabetic tendons, suggesting an initial lag in the organisation (uncrimping) of collagen fibres. (g) However, the elastic modulus within this range does not suggest a significant difference in stiffness. (h) Finally, the strain at 0.3 MPa (physiological maximum stress generated by a muscle) does appear to be significantly higher in the diabetic tendons compared to wild type. wt (*n* = 6), *db*/*db* (*n* = 8). *P < 0.05, **P < 0.01.

When the Achilles tendon was loaded to failure (Figure 3a), the diabetic tendon presented with a significantly lower elastic modulus (13.3 ± 5.2 vs. 29.3 ± 9.7 MPa, *t*_12_ = 4.025, *P* = 0.002, Figure 3b) and a significantly lower ultimate tensile stress (2.13 ± 1.00 vs. 5.15 ± 1.4 MPa, *t*_12_ = 4.737, *P *< 0.001, Figure 3c). There was, however, no significant difference in the failure strain of the diabetic tendons, compared to control (0.296 ± 0.076 vs. 0.326 ± 0.037, respectively, *t*_10.624_ = 0.948, *P* = 0.364, Figure 3d). While these material properties reflect global tissue properties, they are taken at measurement ranges that may not reflect the in vivo functional range. Therefore, we have concentrated on the lower range of the stress–strain curves (Figure 3e), where in vivo stresses generated by the TRI muscle group are likely to occur, specifically the toe region up to 0.3 MPa, which is the maximum isometric stress generated by skeletal muscle (Kissane & Askew, 2024; Matson et al., 2012). Here we see that the diabetic tendons have, on average, a significantly lower stress at this toe region (2.5 ± 0.9 vs. 1.3 ± 0.9 MPa, *t*_12_ = 2.352, *P* = 0.037, Figure 3f). While the elastic modulus does not significantly differ between the control and diabetic tendons at the 0.2–0.3 MPa physiological range (11.8 ± 2.6 vs. 9.7 ± 2.0 MPa, *t*_12_ = 1.730, *P* = 0.109, Figure 3g), the strain of the tendon at this range does significantly differ (0.051 ± 0.008 vs. 0.067 ± 0.014, *t*_12_ = −2.387, *P* = 0.034, Figure 3h).

**FIGURE 4:**
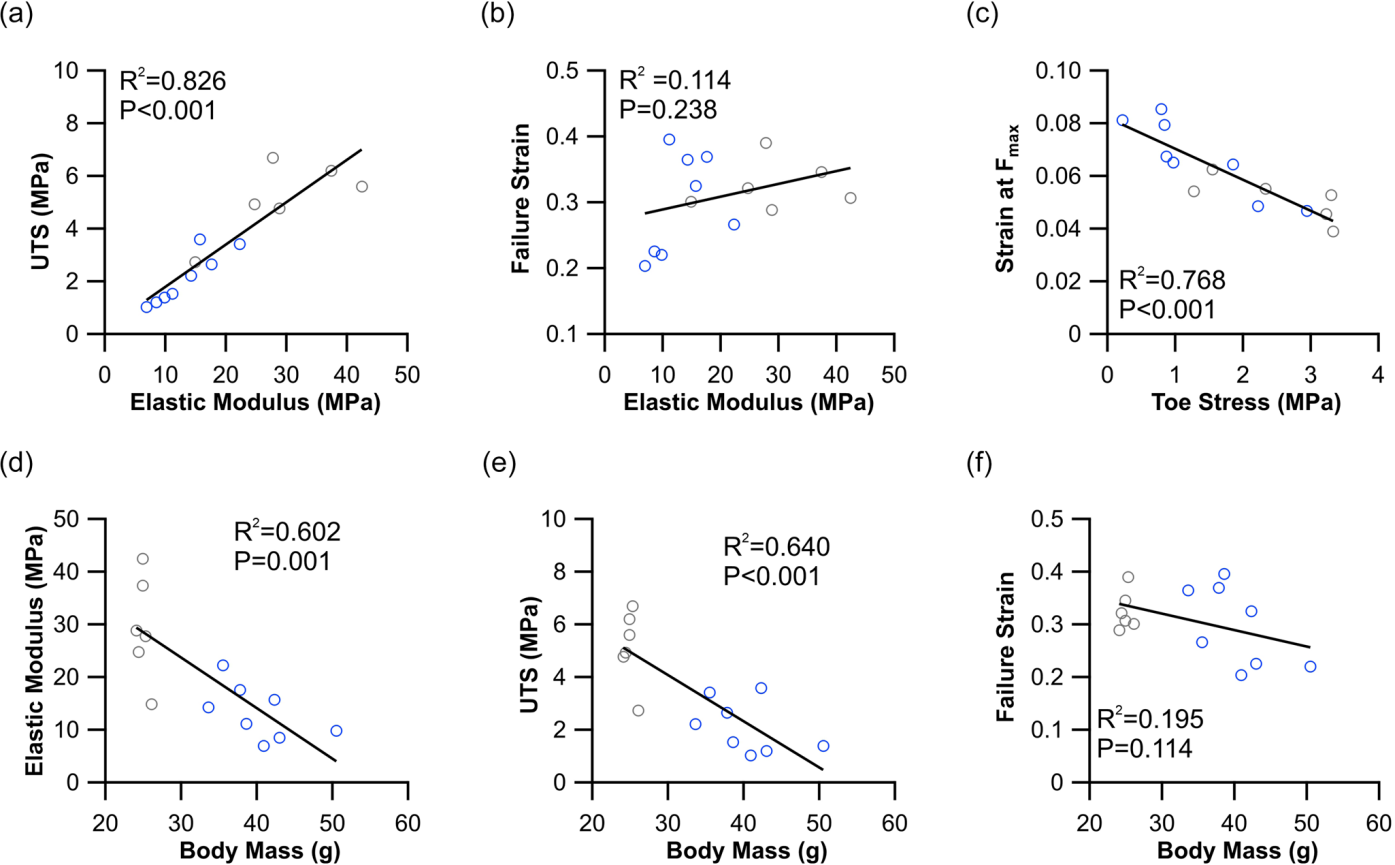
Relationship between body mass and tendon function. (a) Least square regressions highlight the strong relationship between elastic modulus and ultimate tensile strength (UTS). (b) Failure strain did not show any significant relationship with elastic modulus. (c) Similarly, the strain at 0.3 MPa (physiological maximum stress generated by a muscle) appears to be significantly related to the stress at 2% stretch (toe stress). (d, e) There appears to be a significant relationship between tendon elastic modulus (d) and UTS (e) with body mass. (f) However, no significant relationship appears to exist with failure strain. wt (grey circles, *n* = 6), *db*/*db* (blue circles, *n* = 8).

It has been previously shown that elastic modulus is tightly linked to UTS, while no significant relationship exists with failure strain (LaCroix et al., 2013; Matson et al., 2012). Here, we show that this inherent behaviour of tendons is retained in the diabetic tendon (*R* ^2^ = 0.826, *P *< 0.001, Figure 4a,b). Interestingly, we show a similarly significant relationship between the strain at physiological maximum isometric stress (0.3 MPa) and the stress throughout the toe region of tendon elongation (*R* ^2^ = 0.768, *P *< 0.001, Figure 4c). Uniquely, the mechanical properties of these tendons appear to be tightly related to body mass, with a significant decline in elastic modulus (*R* ^2^ = 0.602, *P *< 0.001, Figure 4d) and UTS (*R* ^2^ = 0.640, *P *< 0.001, Figure 4e) as body mass increases. There remains, however, no significant relationship between failure strain and body mass (*R* ^2^ = 0.195, *P* = 0.114, Figure 4f).

### 3.3 Elevated body mass and modified tendon mechanical properties result in altered musculotendon dynamics during trotting

**FIGURE 5:**
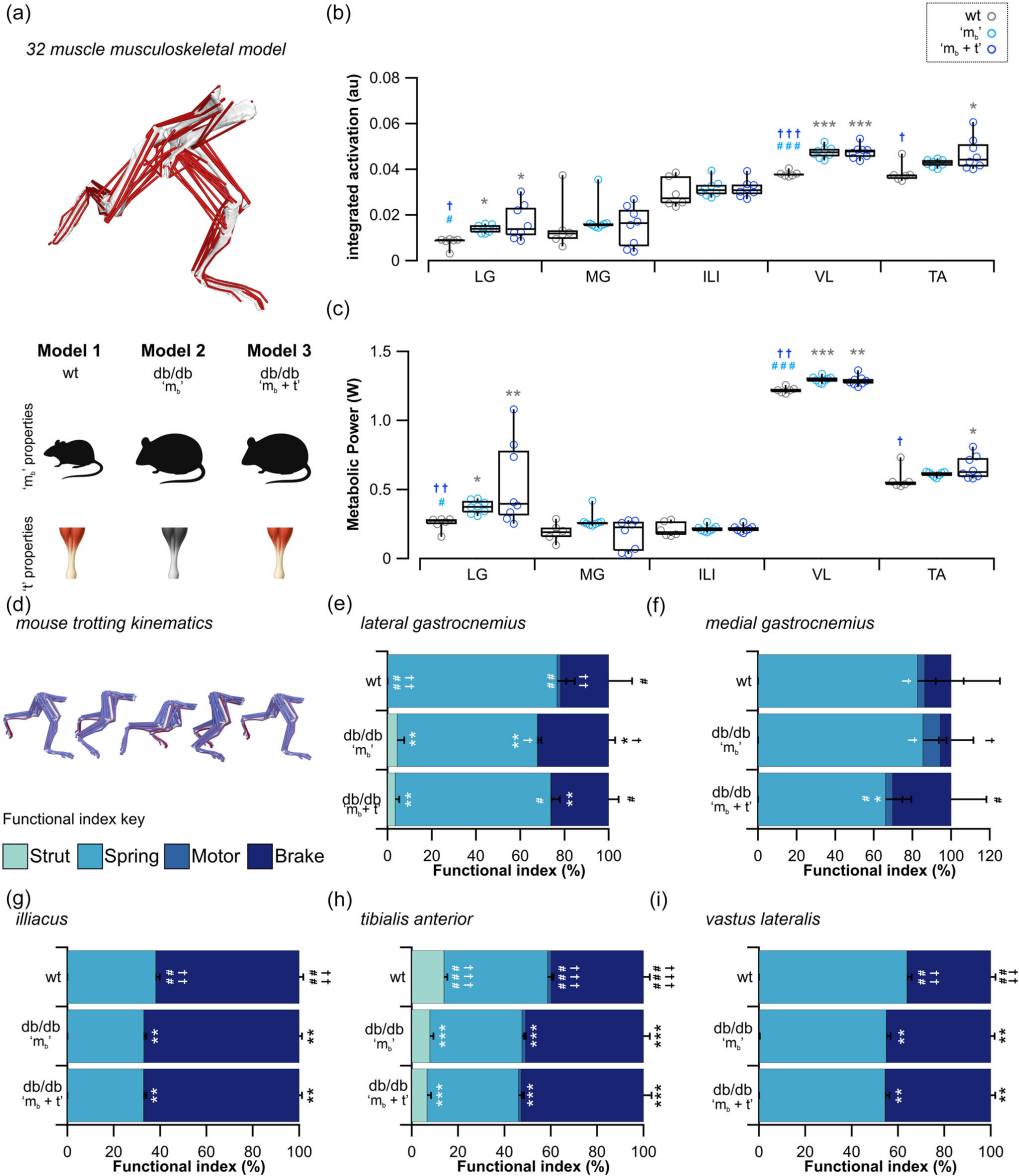
Impact of diabetes on locomotor function. (a) Mouse‐specific musculoskeletal models and simulations were used to predict changes in musculotendon function in response to altered tendon properties. These models comprised control body mass (m_b_) and mouse‐specific tendon (t) material properties (wt), an obese model which comprised the elevated m_b_, without changing tendon material properties (*db*/*db* ‘m_b_’), and a fully diabetic model comprising mouse‐specific m_b_ and tendon material properties (*db*/*db* ‘m_b_ + *t*’). (b, c) Increased body mass and decreased tendon stiffness increased skeletal muscle recruitment levels (b) and the metabolic output (c). (d) During trotting, muscle–tendon unit functional indices were quantified and broadly describe their function as a strut, spring, motor or brake. (e–i) The functional indices are presented for the lateral gastrocnemius (e), medial gastrocnemius (f), illiacus (g), tibialis anterior (h) and vastus lateralis (i). wt (*n* = 6), *db*/*db* ‘m_b_’ (*n* = 8), *db*/*db* ‘m_b_ + t’ (*n* = 8). *P < 0.05 vs. wt, #*P *< 0.05 vs*. db*/*db* ‘m_b_’, †*P *< 0.05 vs*. db*/*db* ‘m_b_ + t’.

Here, we built mouse‐specific musculoskeletal models that reflected the body mass and tendon material properties measured across our control and diabetic cohort of mice (Figure 5a). Having elevated body mass and altered tendon material properties results in a significant increase in muscle activation of the LG (*H*_2_ = 10.522, *P* = 0.005, Figure 5b; 0.017 ± 0.008 vs. 0.008 ± 0.002; *P* = 0.012), VL (*F*_2,19_ = 32.238, *P *< 0.001, Figure 5b; 0.048 ± 0.003 vs. 0.038 ± 0.001; *P *< 0.001) and TA (*H*_2_ = 7.193, *P* = 0.027, Figure 5b; 0.047 ± 0.007 vs. 0.038 ± 0.004; *P* = 0.026) muscles compared to control. Subsequently, there was a significant increase in the estimated metabolic power output in the LG (H_2_ = 10.575, *P* = 0.005, Figure 5c; 0.54 ± 0.3 W vs. 0.25 ± 0.05 W; *P* = 0.009), VL (*F*_2,19_ = 13.938, *P *< 0.001 Figure 5c; 1.29 ± 0.04 W vs. 1.22 ± 0.02 W; *P* = 0.001) and TA (*H*_2_ = 6.662, *P* = 0.036, Figure 5c; 0.66 ± 0.08 W vs. 0.57 ± 0.08 W; *P* = 0.037) compared to controls. Overall, diabetes results in substantial shifts in functional profiles across the musculoskeletal system (Figure 5e–i), with trends towards musculotendon units functioning less like springs and more brakelike.

## 4 DISCUSSION

Obesity is a major driver of T2DM and a key contributor to the locomotor and exercise intolerance that limits the very intervention most effective for disease management. Understanding the mechanisms that lead to impaired exercise function may help to develop more effective and feasible therapeutic strategies. Here, we present a comprehensive overview of the changes in mouse tendon material properties in response to T2DM, and developed a physiologically informed mouse musculoskeletal model and simulation of T2DM to uncover the contribution of elevated body mass and changing tendon mechanics on locomotor performance. Uniquely, we show that tendon mechanics are significantly altered at the physiological range, which may be underpinned by impaired collagen uncrimping. The changes in mechanical properties culminate in an increased tendon strain at peak stress, which in our model of trotting appears to significantly increase the recruitment and metabolic power output of functionally important muscles. Recovery of either non‐pathological tendon material properties or reducing body mass may therefore have a substantial impact on locomotor performance and improve exercise tolerance.

### 4.1 T2DM alters hindlimb tendon material properties independent of changes in muscle mass

The impact of T2DM on muscle mass is a complex interaction to unravel, and a comparably difficult metric to quantify. The increasing proportion of fat mass to total body mass makes it difficult to accurately quantify a relative muscle mass for a given subject. Here, we show that the absolute muscle mass of the TRI and TA of the diabetic mice do not significantly differ to those of the control mice (Figure 1). These data recapitulate those of Suriano et al. (2021), who showed that across developmental stages of these diabetic mice, the size of hindlimb muscles did not significantly differ. However, despite the comparable muscle mass between the diabetic and control mice, the CSA of the Achilles tendon was 1.8× larger in the diabetic mice. The increased Achilles tendon CSA in *db*/*db* mice may reflect chronic overload from elevated body mass (adaptive hypertrophy to normalise stress), but it could also arise from diabetes‐related matrix remodelling that increases size without improving material quality. There are conflicting data across rodent diabetic models in this respect, with some reporting a decrease in Achilles tendon CSA (de Oliveira et al., 2011; Silva et al., 2017) and others reporting an increase (Boivin et al., 2014) (Appendix Figure 1E). There is a similarly variable finding in humans, with some suggesting an increase in tendon thickness (Evranos et al., 2015), and others a decrease (Batista et al., 2008), while there is also some evidence of sex differences among T2DM patients (Akturk et al., 2007). There does not appear to be a predictable physiological response of T2DM on tendon thickness, while there is some evidence that the BMI of T2DM patients may relate to tendon thickness (Abate et al., 2012). However, without simultaneous measurements of tendon material properties, the geometry of the tendon does not provide a meaningful insight into function (Zellers et al., 2021a).

Here, we provide an overview of the viscoelastic properties of the diabetic tendon (hysteresis and stress relaxation) and show that, counter to our initial hypothesis (hypothesis 1a), these properties do not significantly differ from the controls (Figure 2). Tendon hysteresis, one of the more widely measured viscoelastic properties in tendons, describes the energy dissipated due to material viscosity and may have an important impact on the efficiency of locomotion (Finni et al., 2013). To our knowledge, we present the first measure of hysteresis in the diabetic rodent tendon (Zellers et al., 2021a), which aligns with the findings from human in vitro experiments (Zellers et al., 2021b). There have been several attempts to measure hysteresis in vivo, with evidence that individuals with T2DM may have greater hysteresis (greater energy dissipation) than non‐pathological individuals (Petrovic et al., 2018). However, there is some uncertainty on the reliability of hysteresis measurements in vivo (Finni et al., 2013).

Much of the work exploring tendon mechanical properties derives material properties from the linear range, with its extreme stress values that may never be experienced by a tendon in vivo. For example, de Oliveira et al. (2011) show that elastic modulus decreased in the Achilles tendon of the diabetic rat (as we have here supporting our hypothesis 1b; Figure 4a,b), yet these measurements are taken at positions on the stress–strain curve that do not reflect forces produced during locomotion (Eng et al., 2019; Konow et al., 2020). The functionally relevant range of these tissue mechanical properties is often not studied (Lake et al., 2023). Lancaster et al. (1994) showed that the effect of diabetes on tendon stiffness was only significantly detectable within the physiological range, and not at the linear range in the dog patellar tendon. Uniquely, here we show that, at the toe region of this stress–strain curve, the diabetic tendons present with a reduced rate of stress development, suggesting an initial lag in the organisation (uncrimping) of collagen fibres (Gathercole & Keller, 1991; Shah et al., 1979). Subsequently, this lag in uncrimping leads to an increase in tendon strain at maximum in vivo stresses of 0.3 MPa (Kissane & Askew, 2024; Matson et al., 2012). These data align with measures made in the Achilles tendon of humans with T2DM (Evranos et al., 2015; Guney et al., 2015).

### 4.2 Altered tendon material properties and elevated body mass have a compounding effect on locomotor performance

Here, we have uncovered several unique relationships between tendon material properties and body mass. Firstly, the T2DM tendons present with a reduction in elastic modulus and UTS, maintaining this inherent physiological link (Figure 4a) (LaCroix et al., 2013; Matson et al., 2012). The underlying structural basis for this coupling is still unknown, but it is interesting to note that this relationship is not abolished by pathological remodelling, giving hope for a therapeutic potential to reverse this decline. In addition, we see that both elastic modulus and UTS appear to be strongly related to body mass (Figure 4d,e). This, combined with the changes in tensile material properties, means that the diabetic mice are functioning with a severely reduced safety factor (Ker et al., 1988; Pollock & Shadwick, 1994) and, consequently, may be more susceptible to injury.

The importance of these concomitant changes (i.e., in body mass and tendon material properties) on the locomotor performance is unknown, and understanding the consequences of these changes in morphological and mechanical properties will help future targeted therapeutic interventions. It has been previously shown that, in humans, cost of transport increases in T2DM patients (Petrovic et al., 2018), with some suggestion that this is due to increased stiffness of tendons. However, as discussed above, we have shown that in the diabetic mouse (and some human studies; Evranos et al., 2015; Guney et al., 2015), tendons do not appear to be stiffer, but rather more compliant and weaker relative to body mass, given the lower UTS. To predict the functional impacts of these changes in tendon properties, we modified a musculoskeletal model of the mouse hindlimb (Charles et al., 2018, 2024), incorporating morphological and mechanical properties of the diabetic mouse to explore the impacts on individual muscle function during trotting locomotion. The simulations of trotting locomotion in the diabetic mice showed that, in order to ‘successfully’ trot (i.e., satisfy the experimentally derived joint kinematics and applied ground reaction forces), the diabetic mouse models had to significantly increase muscle activation and metabolic output. These changes occurred alongside a shift to a less spring‐like and more negative work‐dominated brake‐like function in the more tendinous, distal hindlimb muscles such as MG and LG. These data support hypothesis 2, and also echo previous work on the metabolic cost of running in humans in response to changes in tendon stiffness (Bates et al., 2025; Sellers et al., 2010; Uchida et al., 2016). Those data showed that more compliant tendons were not always metabolically advantageous as one would assume, with stiffer tendons allowing muscle fibres to operate on a more optimal portion of their force–length curve and thus consumed less metabolic power compared to when tendon compliance was increased. In our data, the normalised fibre lengths of the MG, LG and TA muscles in the diabetic models show deviations away from the optimised fibre lengths of the control models, particularly in the stance phase (the first part of the gait cycle; Supporting information, Appendix Figure A3). This suggests that similar mechanisms could be contributing to the altered muscle dynamics seen here in the diabetic mice, although the specific relationships between fibre length and cost of transport in mice warrant further investigation.

Importantly, applying the same trotting kinematics to the obese mouse models (increase in body mass but no change in tendon stiffness) showed that the extent of these changes was not wholly due to body mass increases. For instance, while significant differences in model outputs were found between the control and obese models, particularly in the muscles located more proximally in the hindlimb and closer to the whole body centre of mass, more substantial differences were seen between the control and diabetic mice in terms of the outputs of the more distal muscles (e.g. LG and TA activation and power; Figure 5b,c; MG function; Figure 5f).

Therefore, these models suggest that while some functional deficits associated with T2DM may arise purely as a result of an increased body mass, the more substantial effects are likely to be more strongly associated with the pathophysiological impacts on tendon function. Specifically, the significantly increased muscle activation (and subsequently force production) in conjunction with the more compliant but weaker tendons of the diabetic mice would likely culminate in a substantially elevated risk of tendon injury or rupture. This enhanced risk of rupture and impaired tendon healing seen in T2DM (Ahmed, 2016; Nichols et al., 2020) further limits the potential of successful intervention with exercise.

### 4.3 Experimental limitations

Firstly, we have neglected to address any influence of sex on the changes in material properties, having used only male mice throughout our experiments. Future work should consider the inclusion of female cohorts, as it has been suggested that the mechanics and morphology of tendons may differ across sexes (Akturk et al., 2007; Sarver et al., 2017). The second limitation here is that we are unable to discern if the altered tendon material properties are a consequence of the metabolic stress associated with diabetes or a physiological consequence of having a significantly elevated body mass. It would be prudent to explore a comparable experimental approach as we present here, using the leptin‐deficient (*ob*/*ob*) mouse line (Giesbertz et al., 2015). These mice present with an obese phenotype, with comparable body mass and adipocyte morphology to the diabetic mice (*db*/*db*) used here (Suriano et al., 2021). However, they do not present with impaired glucose handling and elevated inflammatory tone in their subcutaneous adipose tissue, like their diabetic counterpart (Suriano et al., 2021), which may drive some of the structural and functional remodelling (Lekkala et al., 2019) presented here. However, the inclusion of a cohort of obese biomechanical models tentatively uncovered the cumulative consequence of elevated body mass (biomechanical model 2, obesity alone) and the impact of changing tendon material properties (biomechanical model 3, obesity with altered tendon material properties) on locomotor performance.

There are also limitations associated with the mouse hindlimb model (some of which have been discussed previously; Charles et al., 2018, 2016, 2024), particularly with regard to using it to simulate locomotion in pathological mouse strains, which limit its immediate utility as a preclinical tool. Most obviously, our methodology here of using previously obtained hindlimb kinematics from wild‐type mice to predict muscle dynamics and functions of diabetic mice may not be biomechanically or physiologically accurate. However, as *db*/*db* mice are largely sedentary and rarely trot, gathering such data from these mice would be challenging. Instead, we used these models to provide a platform to perform a theoretical ‘what‐if’ experiment, where the theoretical impacts of T2DM‐associated tendon pathologies can be explored in a way that cannot be done so experimentally. Therefore, these results should not be taken as definitive descriptions of diabetic mouse musculotendon function, but rather broad explorations of the potential impacts of T2DM pathophysiology. While it is likely that the T2DM mice have grossly different kinematics, the fundamental differences in muscle recruitment and function reported here are likely to be upheld (Bates et al., 2025; Sellers et al., 2010). Furthermore, while the assumption of conserved muscle force‐generating properties, particularly fibre lengths, between the control and diabetic mice was based on the similarity in muscle mass between the strains (Figure 1b), it is possible that other aspects of diabetic mouse muscle anatomy or physiology could differ more significantly. These include possible alterations in the phenotype, force‐length relationships or passive stiffness properties of the muscle fibres, which were not incorporated into these models but which could further improve these predictions of muscle function and increase the validity of this mouse hindlimb model as a potential pre‐clinical tool. While these assumptions would preclude these diabetic models from immediate use as preclinical tools, the consistency in these properties between the control and diabetic models allowed us to isolate the impacts of tendon mechanics and thus increase our general understanding surrounding how changes in tendon stiffness impact muscle dynamics.

### 4.4 Conclusion

We showed there to be no detectable changes in the viscoelastic properties of the diabetic tendon. The most functionally relevant mechanical deficit appeared during tensile testing at the low‐stress range (toe region) of the tendon stress–strain relationship, where collagen fibres begin to uncrimp themselves. This impaired function results in a substantially greater demand on the skeletal muscles to generate force, causes fibres to operate outside of their optimal working range, and thus increases their metabolic power output. Therapeutic treatment strategies should look to initially recover non‐pathological tendon material properties to lessen the burden on the skeletal muscles and alleviate their exercise intolerance.

## AUTHOR CONTRIBUTIONS

Conceptualisation, James P. Charles, Brendan Geraghty and Roger W. P. Kissane; Methodology, James P. Charles, Estella Chen, Jeff Hart, Andrea Bell, Brendan Geraghty and Roger W. P. Kissane; Data Collection; James P. Charles, Estella Chen, Jeff Hart, Andrea Bell, Brendan Geraghty and Roger W. P. Kissane Formal Analysis, James P. Charles and Roger W. P. Kissane; Writing—Original Draft, Roger W. P. Kissane; Writing—Reviewing & Editing, James P. Charles, Estella Chen, Jeff Hart, Andrea Bell, Brendan Geraghty and Roger W. P. Kissane. All authors have read and approved the final version of this manuscript and agree to be accountable for all aspects of the work in ensuring that questions related to the accuracy or integrity of any part of the work are appropriately investigated and resolved. All persons designated as authors qualify for authorship, and all those who qualify for authorship are listed.

## CONFLICT OF INTEREST

None declared.

## FUNDING INFORMATION

None.

## Supporting information



Appendix Figures A1–A3.

Data for the figures.

## Data Availability

Data are available upon reasonable request.
