# Supplementary material for: The impact of type 2 diabetes on tendon material properties and locomotor function in the db/db mouse
Source: Exp Physiol. 2026 Feb 15;111(4):2014–25. doi: 10.1113/EP093650 (PMC13140546; doi:10.1113/EP093650)
Supplement: Supplementary file 1 — Appendix Figures A1–A3. [file EPH-111-2014-s002.docx]

# The impact of type 2 diabetes on tendon material properties and locomotor function in the *db/db* mouse.

James P Charles^1^, Estella Chen^1^ Jeff Hart^2^, Andrea Bell^2^, Brendan Geraghty^1^ and Roger W P Kissane^1,3*^

^1^ Department of Musculoskeletal & Ageing Science, University of Liverpool, The William Henry Duncan Building, 6 West Derby Street, Liverpool L7 8TX, UK

^2^ Cica Biomedical Ltd, Knaresborough, North Yorkshire, HG5 9AY, UK

^3^ School of Biomedical Sciences, University of Leeds, UK

* Correspondence to [r.kissane@liverpool.ac.uk](mailto:r.kissane@liverpool.ac.uk)

**Funding:** n/a

**Acknowledgements:** n/a

**Running Title:** The impact of diabetes on locomotor function

**Key words:** Tendon material properties, Musculoskeletal Model, Kinematics, Diabetes Mellitus,

# Data Availability

Data is available upon reasonable request.

# Declaration of Interests

The authors declare no competing interests.

# Author Contribution

Conceptualisation, J.P.C, B.G and R.W.P.K; Methodology, J.P.C, E.C, J.H, A.B, B.G and R.W.P.K.; Data Collection; J.P.C, E.C, J.H, A.B, B.G and R.W.P.K. Formal Analysis, J.P.C and R.W.P.K; Writing – Original Draft, R.W.P.K; Writing – Reviewing & Editing, J.P.C, E.C, J.H, A.B, B.G and R.W.P.K.

**
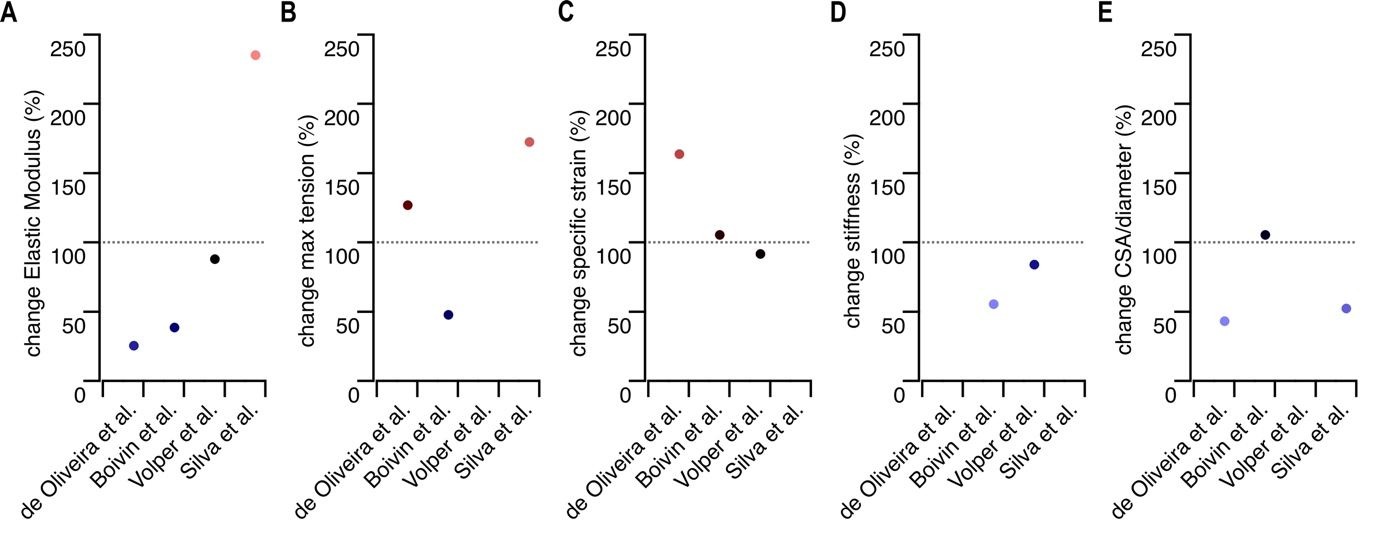
**

**Appendix Figure 1. Changes in material properties of the rodent Achilles tendon.** Data taken from previous studies (de Oliveira *et al.*, 2011; Boivin *et al.*, 2014; Volper *et al.*, 2015; Silva *et al.*, 2017) presented as the change in diabetic tendon relative to their control. Data highlight the variability in findings for changes in Elastic modulus (A) and maximum tension (B). Specific strain (C) appears to generally increase in the diabetic tendon, while the stiffness appears to decrease (D). Overall, there is no clear interpretation for changes in tendon cross-sectional area/diameter (E).

**
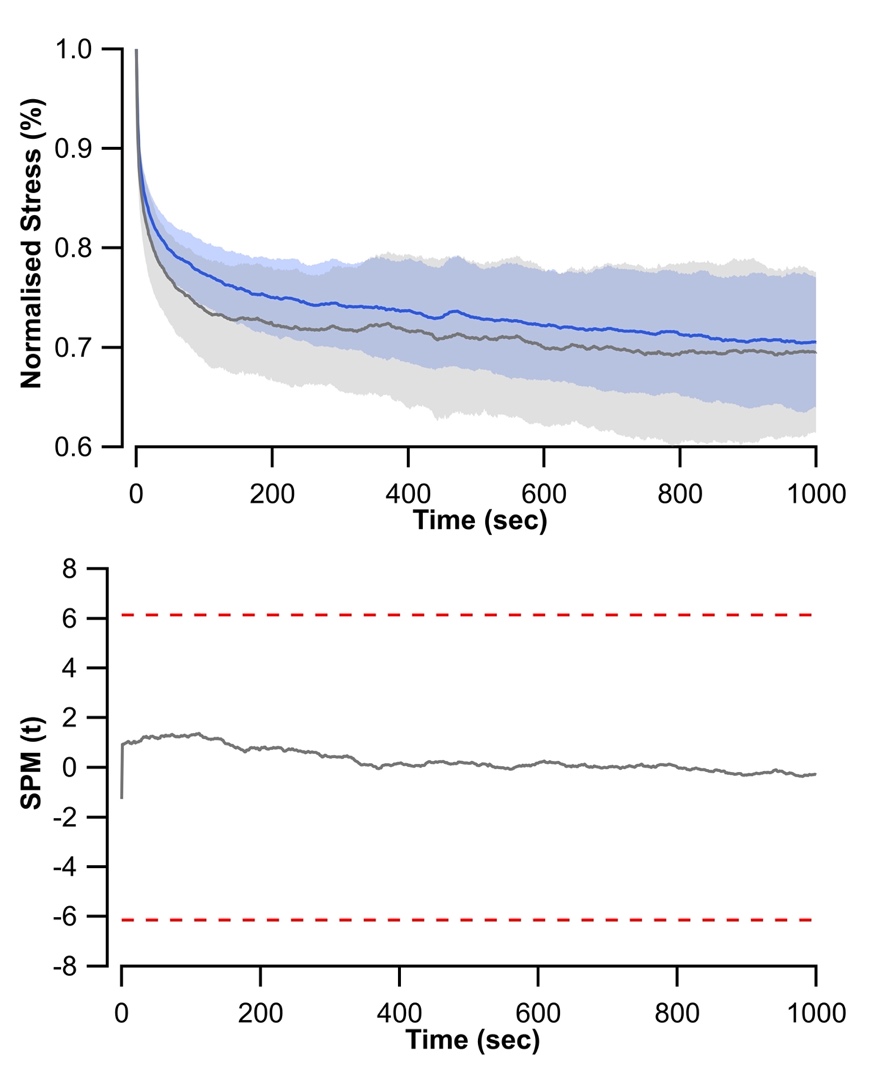
**

**Appendix Figure 2. One-Dimensional statistical parametric mapping of tendon relaxation.** Stress-relaxation experiments show that across a 1000s hold there was no significant difference in the decline in stress, as determined by one-dimensional statistical parametric mapping (1D-SPM) (Pataky, 2012). Red lines indicate the threshold for significance.

**
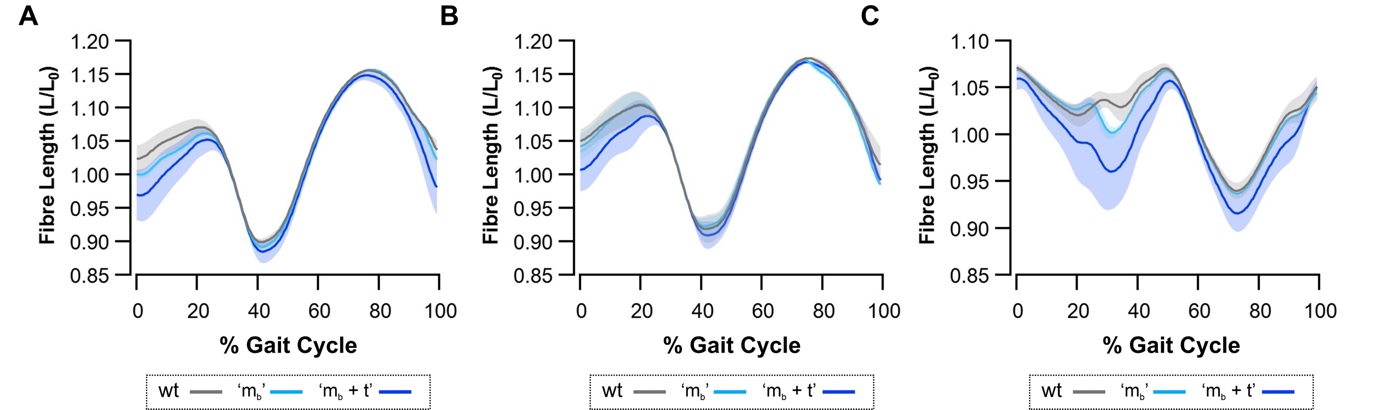
**

**Appendix Figure 3. Fibre lengths during trotting.** Normalised gait cycle fibre excursions for the lateral gastrocnemius (A), medial gastrocnemius (B) and tibialis anterior (C). wt (n=6), *db/db* ‘m_b_’ (n=8), *db/db* ‘m_b_ + t’ (n=8).

# References

Boivin GP, Elenes EY, Schultze AK, Chodavarapu H, Hunter SA & Elased KM (2014). Biomechanical properties and histology of db/db diabetic mouse Achilles tendon. *Muscles Ligaments Tendons J* **4,** 280-284.

de Oliveira RR, de Lira KDS, de Castro Silveira PV, Coutinho MPG, Medeiros MN, Teixeira MFHBI & de Moraes SRA (2011). Mechanical Properties of Achilles Tendon in Rats Induced to Experimental Diabetes. *Annals of Biomedical Engineering* **39,** 1528-1534.

Pataky TC (2012). One-dimensional statistical parametric mapping in Python. *Computer methods in biomechanics and biomedical engineering* **15,** 295-301.

Silva RTB, Castro PVd, Coutinho MPG, Brito ACNdL, Bezerra MA & Moraes SRAd (2017). Resistance jump training may reverse the weakened biomechanical behavior of tendons of diabetic Wistar rats. *Fisioterapia e Pesquisa* **24,** 399-405.

Volper BD, Huynh RT, Arthur KA, Noone J, Gordon BD, Zacherle EW, Munoz E, Sørensen MA, Svensson RB & Broderick TL (2015). Influence of acute and chronic streptozotocin-induced diabetes on the rat tendon extracellular matrix and mechanical properties. *American Journal of Physiology-Regulatory, Integrative and Comparative Physiology* **309,** R1135-R1143.
